# 146. Review of Urine Culture Screening Practices Prior to Urologic Procedures

**DOI:** 10.1093/ofid/ofad500.219

**Published:** 2023-11-27

**Authors:** Anthony J Jeong, Sessen Dudek, Jessica G Bennett, Neena Thomas-Gosain

**Affiliations:** University of Tennessee Health Science Center, Memphis, Tennessee; University of Tennessee Health Science Center, Memphis, Tennessee; VAMC Memphis, Germantown, Tennessee; University of Tennessee Health Science Center, Memphis, Tennessee

## Abstract

**Background:**

Screening for UTIs prior to urologic procedures is a well-established practice. However, screening for asymptomatic bacteriuria remains more controversial, especially for procedures considered to be low-risk for complications. The aim of this study was to characterize current screening practices at our facility.

**Methods:**

This retrospective study reviewed outpatient urologic procedures performed between 10/01/2022 and 12/31/2022. Procedures were stratified by risk based on current American Urological Association guidance. Charts were reviewed for the following information: urinary symptoms, ucx results, antibiotic prescriptions, post-op complications. The primary outcome was the incidence of urinary (GU) infection or infection related hospitalization (IRH) within 30 days post-procedure. Secondary outcomes include ucx screening rates and risk of infection based on presence of urinary symptoms.

**Results:** 210 procedures were reviewed. Risk of GU infection ranged from 8.2% to 12.9%, with the highest rates seen in intermediate- and high-risk procedures. Risk of IRH increased by increasing level of risk (Table 1).

Pre-op ucx were ordered in 35% of procedures, however frequency of ordering varied based on level of risk, with intermediate-risk (96%) much more likely to have been screened and no screening was done prior to high-risk procedures (Table 2).

Symptomatic patients were more likely to have a pre-op ucx (60.4% vs 28.2% if asymptomatic). Ucx positivity was 3-fold higher if patient was symptomatic. Rates of GU infection and IRH varied based on presence of symptoms and whether preop ucx was ordered. (Table 3)

**Table 1:**
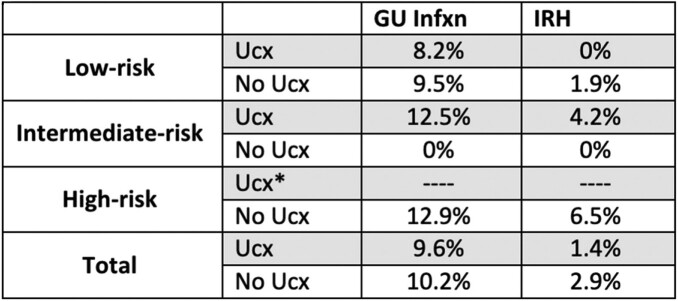
Risk of GU infection or IRH by ucx ordering, stratified by risk *no data since no ucx were ordered in high-risk group

**Table 2:**
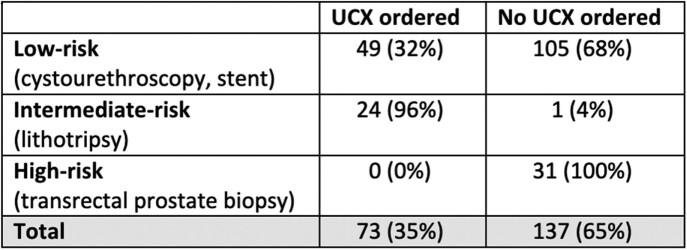
Preoperative ucx ordering by risk stratification

**Table 3:**
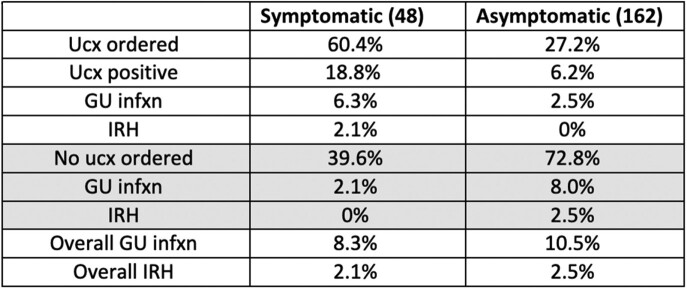
Ucx screening by symptom status

**Conclusion:**

In this study sample, risk of infectious complications (especially IRH) increased with increasing level of procedural risk. However, screening trends were inconsistent limiting conclusions about the impact of ucx on the incidence of infectious complications prior to intermediate and high-risk procedures.

The rate of ucx screening was higher when urinary symptoms were present, but clear trends did not appear. This study highlights the need for consistent screening practices, primarily based on risk of procedure. Larger numbers may help answer the question of whether or not screening should be based on presence of symptoms.

**Disclosures:**

**All Authors**: No reported disclosures

